# Molecular epidemiology and pathogenic potential of underdiagnosed human papillomavirus types

**DOI:** 10.1186/1471-2180-8-112

**Published:** 2008-07-04

**Authors:** Stefano Menzo, Andrea Ciavattini, Patrizia Bagnarelli, Katia Marinelli, Stefano Sisti, Massimo Clementi

**Affiliations:** 1 Institute of Microbiology, Università Politecnica delle Marche, Ancona, Italy; 2 Institute of Obstetrics and Gynecology, Università Politecnica delle Marche, Ancona, Italy; 3 Institute of Pathology, Università Politecnica delle Marche, Ancona, Italy; 4 Institute of Microbiology, Università Vita-Salute San Raffaele, Milano, Italy

## Abstract

**Background:**

Human papillomavirus (HPV) tests are crucial diagnostic tools for the prevention of neoplastic lesions of the uterine cervix. However most commercial methods are designed to detect high-risk (HR) HPV types and a limited selection of low-risk ones, thus missing a fair number of intermediate/low-risk types. As a result, many HPV infections remain undiagnosed, generating distrust in virological diagnosis among gynaecologists, who continue to rely preferentially on cytological and colposcopic findings.

**Results:**

In this study, we tested 6,335 consecutive clinical samples, most of them from Italian patients with cytological abnormalities. The samples, collected in 2000–2007, were analyzed using PCR amplification of a 173–206 bp (depending on HPV type) conserved region in the L1 open reading frame, restriction endonuclease analysis and, where required, sequence analysis for type determination. Analysis of a smaller male sample and long term follow-up of a few female subjects was also performed. A total of 2,161 samples tested positive for HPV DNA (32.1%); 21.3% of them were mixed infections. Overall, 59 known and 2 unknown HPV types were detected. Their relative prevalence was calculated; notably, types not clearly identifiable using the most common commercial method accounted for 36% of infections. Clinical findings associated with the underdiagnosed types ranged from H-SIL to low-grade abnormalities, although none of these infections resulted in invasive cancer.

**Conclusion:**

Given the high prevalence of some underdiagnosed HPV types in the population (principally HPV53, HPV66, HPV84, and HPV87) and their frequent association with cytological abnormalities, techniques capable of detecting and typing them would prove extremely useful.

## Background

Human papillomavirus (HPV) infections of the genital tract are highly prevalent in the population worldwide; indeed, it is estimated that the great majority of sexually active individuals become infected with one or more of these viruses in their lifetimes. Most conditions are transient and are cleared without consequences. However, in a small proportion of cases, infections associated with the so-called high-risk (HR) HPV types can persist in typical lesions with a high viral load for years, and a fraction of such lesions eventually progress to invasive malignancies. Although many cofactors normally concur in this process, the HPV type implicated (the type-specific viral oncogenes) and the inability of the immune system to clear the infection are by far the most important determinants of HPV-related diseases (for review see: ref [1]). Recent work by the IARC (International Agency for Research on Cancer) network has contributed to clarify the role of HPV genotypes [2,3], and has assigned to each HPV type an Odds Ratio for the risk of cancer development. This information is crucial for prognosis and correct management of infected individuals. From a diagnostic and clinical perspective, HPV typing appears to be as important as HPV DNA detection. Several diagnostic tests for HPV DNA detection and typing are available. Some are based on PCR amplification of HPV DNA using primers recognizing a conserved sequence of the viral L1 region, followed by typing using restriction endonuclease polymorphism, solid phase hybridization with type-specific probes, or sequencing of the amplified product [4-7]. Other assays are based on liquid-phase hybridization without previous template amplification, like the only FDA-approved test for HPV DNA detection, Hybrid Capture 2 (HC2; Digene/Quiagen, Gaithesburgh, MD, USA), which detects a selected number of types, i.e. low-risk types 6, 11, 42, 43, and 44, and HR types 16, 18, 31, 33, 35, 39, 45, 51, 52, 56, 58, 59 and 68. Finally, assays like INNO-LiPA [8] (Innogenetics, Gent, Belgium) and Amplicor/LA (Roche Molecular Systems, Alameda, CA, USA) use hybridization (after PCR amplification) with a variable number of probes organized into arrays for detection and typing. The diagnostic approach to HPV infections is thus strongly affected by the choice of diagnostic strategy, and a number of types fail to be detected in the vast majority of virology laboratories worldwide. As a consequence, the pathogenic potential of a large proportion of mucosal HPV types cannot be correctly evaluated and the possible changes in the molecular epidemiology of  HPVs cannot be adequately monitored.

We investigated the distribution of underdiagnosed HPV types in genital infections, using a molecular strategy capable of detecting a large number of known (and possibly unknown) HPV types, and calculated their relative prevalence and their role in the arising of cytological abnormalities and dysplastic lesions.

## Results

### Detection and typing of HPV DNA in samples from female patients

**Figure 1:**
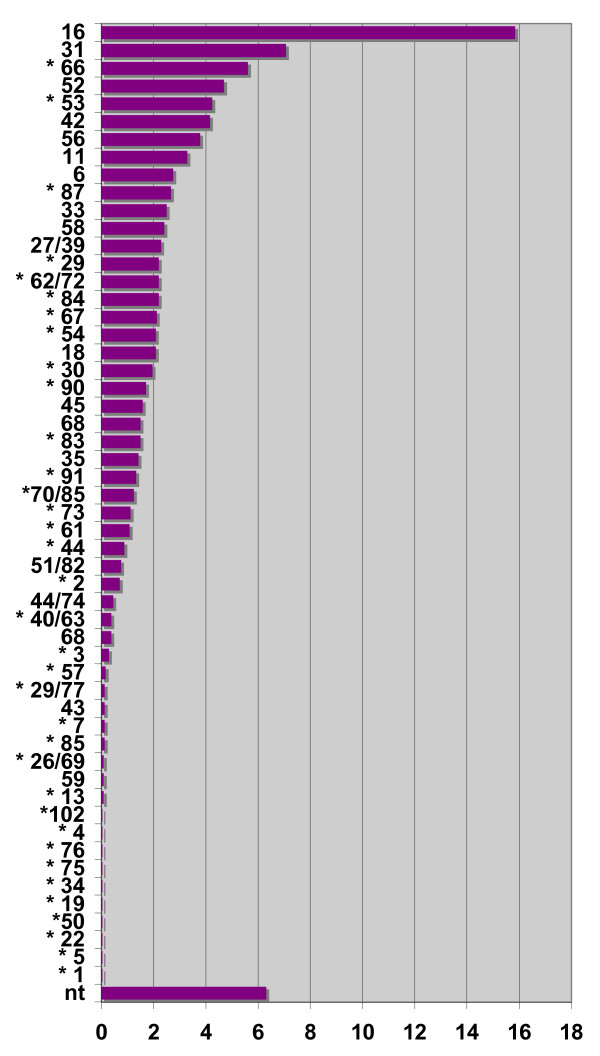
**Global relative prevalence of HPV types**. Relative prevalence of HPV types among 2,110 positive female specimens (years 2000–2007) expressed as percentage of total HPV infections (in multiple infections, each infection is counted separately). Infections not typed by the assay used in this work: nt. Asterisks mark HPV types not typeable by the Hybrid Capture 2 assay. 44 (55): subtype of HPV44 formerly classified as HPV55.

A total of 6,029 female genital samples (including 47 biopsies) received from 7 different clinical centres in 2001–2007 were analyzed for HPV DNA; 2,110 (33.8%) tested positive for HPV DNA. A total of 54 different restriction patterns were detected, identifying up to 62 different known types; of these, 46 unique restriction patterns identified single types, while 8 restriction patterns identified two different types each. Interestingly, most of the latter patterns identified couples of HPV types phylogenetically related within the same species (29/77, 44/74 and 62/72 among low-risk, and 26/69, 51/82 and 70/85 among ones), while in two cases they identified pairs made up of two more distantly related types: 27/39, a low-risk cutaneous/mucosal and a high-risk type and 40/63, a low risk mucosal and a cutaneous type. In the case of mixed infections, the individual co-infecting types were mostly resolved by the analyses of the mixed restriction patterns, up to four different types. In cases where restriction analysis of the amplified product resulted in a clear but unknown pattern, the amplification product was sequenced to achieve a definitive result, mostly showing the presence polymorhisms in the restrictions sites. Most of these variants are extremely rare, while common variants were detected for HPV type 66 and 54. Nonetheless, some mucosal HPV types were never detected in our samples, including HPV32, HPV71, HPV73, HPV86 and the most recently characterized types from HPV97 to HPV106 (except HPV102, which was detected once). A few exclusively cutaneous types, i.e. types 1, 4, 5, 8, 19, 22, 50, 75, and 76, were single findings. The source of these samples is unknown, possibly they derive from warts in the groin area. The frequency distribution of the types detected in the present study is summarized in Figure 1. As in most other settings, HPV16 was the most prevalent virus, followed by other types: HPV31, HPV66 and HPV52. Not surprisingly, given that sampling was driven mostly by cytological abnormalities rather than by the presence of condylomata, the most prevalent true low-risk type (HPV42) was the 6^th ^most frequent type. According to the technique used, 21% of all fully typed infections were mixed, 17% were double, 3% were triple and less than 0.2% were quadruple or more. However, the latter figure might be inaccurate, because some multiple infections were untypeable and the precise number of coinfecting viruses could not be determined. In Figure 1 the types marked by an asterisk are those that would not have been correctly identifiable by the HC2 technique (with either a false negative or a wrong type result), accounting for 36–38% of those detected by our method. Similarly, 28–32% and 14–16%, respectively, would not have been identifiable by INNO-LiPA (25 genotype version) and ROCHE LA (37 genotype version). The uncertainty of these figures depends on the fact that some HPVs are typed as couples (as described previously) by our assay, and other assays detect only one type in some of those couples.

### HPV types and lesion grade

**Figure 2:**
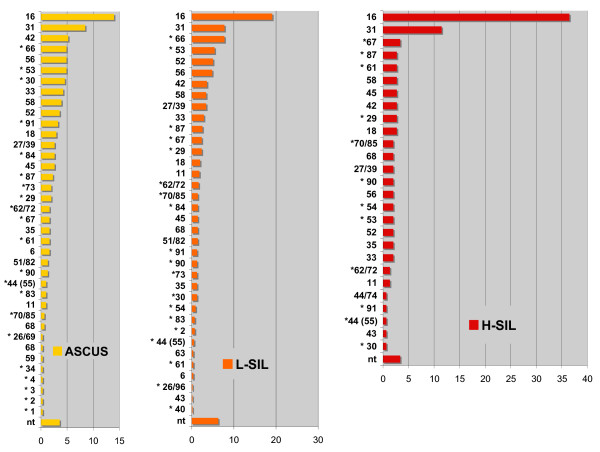
**HPV types in cytological alterations**. Relative prevalence of HPV types associated with three different grades of cytological findings expressed as a proportion of total HPV infections in the three groups. Asterisks mark HPV types not typeable by the Hybrid Capture 2 assay. 44 (55): subtype of HPV44 formerly classified as HPV55.  ASCUS, n = 296 samples; L-SIL, n = 555 samples; H-SIL, n = 143 samples.

Positive samples for which cytological information was available were subdivided into grade of abnormality (ASCUS through H-SIL) and type prevalence was calculated. As shown in Figure 2, the prevalence of high-risk types increased with lesion grade, while the opposite was true for most low-risk types. Although the proportion of HC2-undetectable viruses decreased, these viruses were still found in 31/143 (22%) H-SIL samples (excluding all mixed infections with HC2-detectable viruses) and a few of them, as the main high risk ones, showed a rise in prevalence in H-SIL samples compared to ASCUS, (HPV67: +100%, HPV61: +60%, HPV29/69: +37% and HPV87: +15%), although on the basis of limited numbers. To identify the clinical significance of HC2-undetectable types, we analyzed the associated cytological findings, again excluding the mixed infections with HC2-detectable viruses. Of 289 HC2-undetectable infections for which cytological findings were available, serious abnormalities (H-SILs) were detected in 17 samples (5.9%) (compared with 12.5% of infections with HC2-detectable viruses, i.e. 119/951). In six HC2-undetectable infections the clinical and cytological findings had led to surgery, irrespective of the HPV type involved; however, none of these patients showed CINIII/carcinoma *in situ *(CIS) at histological analysis of the surgical piece.

Finally, in 4 subjects who underwent surgical excision without prior virological testing, but whose lesion was indeed histologically classified as CINIII/CIS, the follow-up genital swabs tested positive only for HC2-undetectable viruses (HPV53 or HPV87) suggesting that these viruses might have been implicated in the pathology observed. However, the analysis of their available formalin-fixed surgical samples revealed HPV16 infection in 3 cases and HPV31 in one, indicating that HC2 undetectable viruses were co- or subsequent infections. This underlines the importance of HPV testing and, in particular, of typing prior to excision, in order to perform the correct prognostic evaluation and a reliable subsequent follow-up.

### HPV DNA in samples from male patients

**Figure 3:**
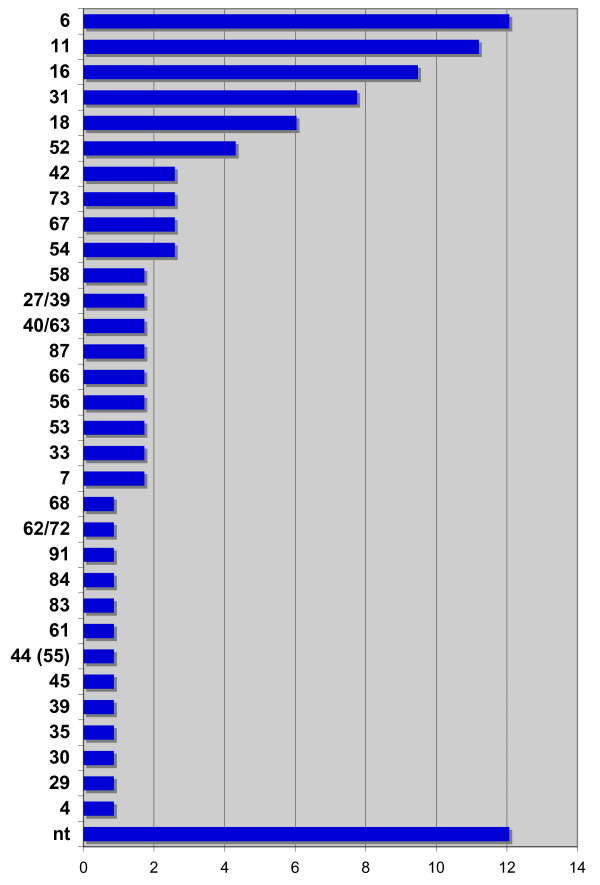
**HPV types in males**. Relative prevalence of HPV types in 103 HPV-positive male genital specimens, expressed as a percentage of total male HPV infections. Asterisks mark HPV types not typeable by the Hybrid Capture 2 assay. 44 (55): subtype of HPV44 formerly classified as HPV55.

Of all the samples analyzed, 306 were genital swabs from male subjects. Clinical information on the reasons leading to HPV DNA testing was available for a minority of these samples, the main two causes being an HPV DNA-positive female sexual partner and detection of condylomata acuminata. Overall, 51/306 samples were positive for HPV DNA (16.5%). Of note, 83/255 (33%) of the samples collected at the glans penis were positive for HPV DNA, as opposed to 4 of the 51 urethral samples (7.8%). This suggests that the urethra is not a major site of replication for most HPV types and that it may not be the ideal site for sampling in male subjects (just as it is not a site for the development of HPV-related tumours [9,10]). Data from 52 positive male samples analyzed before 2001 (from a comparable population and tested by the same method) were added to these 51 cases to enrich the analysis. The overall type distribution is shown in Figure 3. Untypeable infections due to faint bands were more frequent among male than among female samples, suggesting a very low viral load in some of these specimens. The difference with female samples was striking, as low-risk types HPV6 and HPV11 were more prevalent than HPV16, and the whole general pattern was different. This can be attributed to the fact that in males specimen collection was not guided by cytological abnormalities, and therefore, although biased by the fact that the obvious presence of condylomata in males may lead to the request of HPV analysis more frequently than in females, the male sample probably reflects more accurately the general circulation of HPV types in the normal sexually active population.

To gain further insights into the discrepancy observed in type prevalence between the genders, we next tried to identify stable couples where both partners were positive for HPV DNA. We were able to document 26 such couples. Type concordance (including mixed infections with at least one type in common) was very low: 8/26 (30.8%); in all other cases the partners harboured different HPV types, confirming a high turnover of HPV infections in the population. In addition, no correlation could be found between persistence in the female partner after treatment and the presence of same virus in the male partner. The latter finding, despite the small sample, argues against the systematic investigation of male partners, which has lately become fashionable. Moreover, the usually obvious nature (and the low incidence) of dangerous HPV-related lesions in males does not justify widespread virological testing, even in partners of HPV-positive women.

### Long-term virological follow-up of HPV-infected subjects

**Figure 4:**
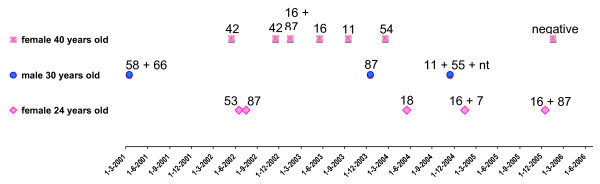
**Multiple subsequent infections**. HPV infections in 3 subjects with multiple subsequent infections during long-term follow-up: HPV DNA testing and typing along the time scale.

The extended observation period by the same method enabled some longitudinal analysis. Although with different schedules, depending on the different clinical centres or practitioners, a significant number of patients were followed-up after the initial sampling. Of these, 157 showed HPV DNA persistence (presence of HPV DNA of any type) in at least 2 consecutive samples, in the absence of treatment. Persistence >2 years was observed in 30 perfectly immunocompetent individuals; in 10 of them the same type infection persisted in the absence of treatment. The HPV types identified in these 10 cases were 31, 52, 53, 54, 56, 61, 66, 84. HPV66 persisted longest (6 years) in one patient, without cytologically (ASCUS) and colposcopically alarming lesions; to our knowledge the virus has not yet been cleared. These data confirm the notion [11] that cervical HPV persistence is not an exclusive feature of the most dangerous types, although in our sample many high-risk infections underwent some sort of treatment and no reliable comparison was possible. The remaining 20 subjects showed consecutive multiple infections with different HPV types. The sequence of the infections was particularly striking in three cases (Figure 4). Despite the lack of information on their risk factors for HPV infection, no reason of impaired immunocompetence was reported in these patients. If we consider all the other patients in follow-up, virtually all high-risk infections were either spontaneously cleared or successfully treated within two years. Treatment was performed in 16,5% of the subjects with HR infections in one of the clinical centres (no data were available from the other centres). None of the over 6000 patients included in this study, undergoing pap-test and virological analysis, was reported to develop invasive cervical cancer, demonstrating the high success rate of correctly implemented prevention based on screening and typing. In the same time period of the study, a few cervical cancers were reported in the clinical centres, but only in patients who underwent no screening; their samples were therefore not included.

## Discussion

Despite the recent progress in the understanding and definition of the risk of cancer associated with the so-called "high risk" HPV infections, the epidemiology and clinical significance of rare HPV infections and those that are not associated with cancer has not been completely clarified. Most data come from studies limited either by sample size or by the inadequacy of the technique applied in detecting and typing a sufficient number of the HPV types circulating in the population. Since all such infections give rise to a range of cytological abnormalities that are readily detected by the Pap smear test, deeper insights into their distribution and role in generating cytological changes could guide clinicians towards aetiology-driven therapeutic approaches, confining invasive treatment to those infections really requiring it. Most clinical laboratories use commercial assays which, though sufficiently sensitive in detecting most high-risk HPV genotypes and other common low-risk ones, fail to identify many infections responsible for cytological abnormalities. We used a PCR amplification method capable of detecting the HPV DNA sequences of a large number of mucosal HPV genotypes, associated with different strategies for typing the amplified viral DNA. Over the past few years, several untyped sequences have been detected at our laboratory using this method. Analysis of the sequences (all deposited in the databases, and published elsewhere) led to the complete sequencing and molecular characterization in our lab of a novel virus that was officially designated *cand*HPV87 [12]. To date, two of the sequences detected in the former study remain untyped (representing either subtypes or potential novel types) but, unlike HPV87, they have not been found again in the population. Using this strategy, we demonstrated a high overall relative prevalence of HPV types not identifiable using the most common commercial methods, accounting for more than one third of all infections harbouring detectable HPV DNA (in the case of HC2). These results are in agreement with those obtained by sequencing in Germany [13], and suggest that this distribution is not restricted to italian patients. This could represent a problem worldwide, since only a minority of facilities use assays that detect most genotypes. The clinical findings associated with these types ranged from H-SILs to (more frequently) low-grade abnormalities, as shown in the present study. Although we could find no link between underdiagnosed HPV genotypes and histologically confirmed CINIII/CIS lesions (but histological data were available only for 22/117 patients from a single clinical centre, no data were available from the other clinical centres), some potentially dangerous infections might remain undetected. In other cases, lesions falsely negative for HPV DNA or with inaccurate typing may generate confusion and/or alarm. Indeed, in some reports (for comprehensive meta-analyses see refs. [2] and [11]) a small, but consistent proportion of malignancies was observed to arise from infections with HPV73 and HPV82, and other authors [14] found a few CINIII/CIS cases associated to underdiagnosed types. In addition, some of these underdiagnosed viruses, i.e. HPV26, HPV30, HPV53, HPV66, HPV67, HPV69, HPV70, and HPV85, are phylogenetically related [15] to the HPVs more frequently associated with cancer. Only a multicentric analysis, based on a very large number of patients, could clarify this aspect.

## Conclusion

Although the present study is limited in terms of the geographical distribution and selection criteria of the sample population, and might not have the solidity of a formal epidemiological study, the sheer number of samples and the extended period in which they have been collected provide firm support for the notion that ideal assays for the diagnosis of HPV infection should detect and type reliably the large majority of HPV types affecting the genital mucosa. Sensitivity to low copy numbers is less important than specificity and range of types identifiable. The most promising technology, capable of providing the necessary information with relative ease of execution and good potential for standardization, is based on macro- or micro-arrays. Current assays using such flexible molecular methods are still limited in terms of number of probes and specificity [16], therefore the development and use of more powerful and reliable arrays should be encouraged (rather than hampered by the protection of intellectual property, as it sometimes happens). Detecting and typing all possible genital papillomaviruses circulating in the human population should also turn out to be a useful aid both for the design and the use of successful preventive (and possibly therapeutic) vaccines.

## Methods

### Patients

Genital samples (swabs and biopsies) from Italian female and male patients were referred to our laboratory for a range of HPV-related genital conditions including cytological abnormalities, colposcopically detected lesions and condylomata. Known HIV-positive or transplant recipient patients were excluded from this study. Overall, 6,335 consecutive clinical samples received in 2000–2007 were evaluated for the presence of HPV DNA. The samples included a small number of fixed bioptic or surgical specimens.

### DNA extraction, PCR amplification, and typing

The technique for DNA extraction and amplification from clinical samples has been described elsewhere [9]. Briefly the MY11 [4] and GP6+ [6] primers (modified and synthesized as follows: sense primer GCA CAG GG(T/A) CAT AA(T/C) AAT GG, antisense primer: AAC TGT AAA TCA (A/T)AT TC(T/C) TC) were used to amplify the L1 conserved region of the HPV genomes. In our hands this combination of primers has proved to be more sensitive and capable of amplifying a broader range of genotypes than both the original MY11/09 and GP5+/6+ pairs. In each extraction/amplification session a negative control sample (plain water) was subjected to extraction and amplification along with the clinical samples, to exclude cross-contamination. Extraction of DNA from fixed samples was performed on 20-μm-thick slices deparaffinized with xylene and hydrated by serial passages in ethanol with growing proportions of water. After hydration, samples were processed like the cytological material. The amplified product (173–208 bp) was subjected to restriction fragment length polymorphism (RFLP) analysis, after digestion with *Rsa*I and *Tru*91 restriction endonucleases, directly applied to an aliquot of the amplification reaction. To resolve cases with similar restriction patterns (HPV 10/27/39/67/70/85, HPV2/30/53 or HPV52/84), the amplified product was also digested with *Afl*III restriction endonuclease. In a limited number of samples restriction analysis did not yield a satisfactory result, due to (in order of frequency): 1) faint bands in the presence of non-specific products, 2) multiple infections with many different types, 3) unknown restriction pattern due to polymorphisms (mostly subsequently resolved by sequencing the amplified product).

### HPV DNA sequencing and sequence analysis

In some of the cases yielding unidentifiable restriction patterns, the amplified product was sequenced by the cycle sequencing technique, followed by detection on an ABI 3100 capillary electrophoresis automated sequence analyzer (Applied Biosystems, Foster City, CA, USA). Sequences were subsequently analyzed using the NCBI BLAST tool and phylogenetic analysis (Phylip package) after multiple alignment (Clustal) of HPV sequences available from public databases.

## Authors' contributions

SM, PB and KM performed some of the sampling and all the restriction and sequencing analyses, SM and MC conceived the study and organized the data, AC selected specific clinical cases, provided clinical data and performed colposcopy and, in a few cases, cytology, SS performed histological analysis. SM drafted the manuscript, with helpful advise by MC and PB.
